# Different information needs—The major reasons for calling the helpline when invited to colorectal cancer screening

**DOI:** 10.1111/hex.13496

**Published:** 2022-04-07

**Authors:** Kaisa Fritzell, Anders Kottorp, Anna Jervaeus

**Affiliations:** ^1^ Department of Neurobiology, Care Sciences and Society Karolinska Institutet Stockholm Sweden; ^2^ Faculty of Health and Society Malmö University Malmö Sweden

**Keywords:** colorectal neoplasms, counselling, endoscopy, knowledge, mass screening, public health

## Abstract

**Introduction:**

This study pertains to the design of a decision aid (DA) to shed light on information and support needs in colorectal cancer screening, with the aim to explore the calling patterns to the Screening of Swedish Colons (SCREESCO) study's helpline.

**Methods:**

A cross‐sectional study was conducted with data from documented telephone calls to the SCREESCO study, including individuals, 59–60 years, randomized to colonoscopy or high sensitive faecal immunochemical test (FIT).

**Results:**

More than 2000 calls (women 58.5%; colonoscopy 59%) were analysed. Calling patterns: unsubscribing from screening, confirmation of participation, logistical concerns about the screening procedure, counselling, and FIT screening difficulties or in need of a new FIT test. Comorbidity was the most frequent reason for unsubscribing and most of the counselling calls included questions about the FIT test or the colonoscopy.

**Conclusion:**

Most of the calls to the helpline seemed to be related to individuals' lack of understanding about the organization of the screening programme and the screening procedure. Therefore, we find it important to further stress the tailoring part in our DA developing process, that is, provide limited information initially, with the possibility of access to more, if desired by the individual, still with respect to the individual's needs, health and digital literacy.

**Patient and Public Contribution:**

Individuals representing the public and invited to SCREESCO participated since we analysed their calls to the helpline. The findings will contribute to our continued work with the DA where the public will contribute and participate.

## 1 INTRODUCTION

Cancer screening programmes in different areas are becoming more and more important, including screening for colorectal cancer (CRC). CRC screening programmes, targeting one of the most common cancers among women and men worldwide, although with geographical variations, are widely spread, especially in high‐income countries.^1^, ^2^ Sweden has, since 2008, an ongoing regional (Stockholm‐Gotland) CRC screening programme with a biennial faecal immunochemical test (FIT) running for all individuals aged 60–69.^3^ This year, based on the national randomized controlled Screening of Swedish Colons (SCREESCO) study (ID: NCT02078804),^4^ Sweden is about to launch a national CRC screening programme region by region.

CRC screening programmes, however, are well known for facing challenges due to low participation. This jeopardizes the benefits of screening, that is, decreased incidence and mortality in CRC^2^, ^5^ and equal access to CRC screening programmes.^6^, ^7^, ^8^, ^9^ However, alongside a high uptake, it is also desired that individuals make an informed decision on knowledge rather than ignorance, misconceptions, or fear, especially since screening programmes approach seemingly healthy individuals.^10^, ^11^, ^12^ To enhance an individual's autonomy while participating in CRC screening, a decision aid (DA) can be helpful.^13^, ^14^ DAs are often web‐based tools with various content, including disease‐related information, information about the procedure (screening in this case), benefits and harms of the procedure and value clarification exercises^13^, ^15^, ^16^ to facilitate what decision best match the individual's values.^11^ Existing evidence shows that people using DAs in connection to a treatment or screening decision increase their knowledge, feel more informed and are certain of what matters most to them.^11^, ^13^ DAs can also have an important role for individuals with lower levels of educational attainment and health literacy since the DA facilitate learning by providing interactive information in different formats and on different levels.^16^, ^17^ This study is part of a larger project aiming at developing a DA in connection to population‐based CRC screening in Sweden. The DA will be based on previous research, within the SCREESCO study, on participation and nonparticipation in CRC screening exploring values and preferences^18^ health literacy,^19^ anxiety,^20^ shared decision‐making^21^ and experiences of the screening procedure itself.^22^

The goal of the DA is to help individuals make an informed screening decision. To be able to evaluate the implementation of the DA, the development process needs to be thorough and systematic, and include knowledge around questions people have when invited to screening. Therefore, we will follow The International Patient Decision Aid Standard (IPDAS) suggested five steps to structure the development process (1/scope, 2/steering group, 3/design, 4/alpha‐ and 5/beta testing).^15^, ^23^ This study aims at exploring the calling patterns to the SCREESCO study's helpline, which pertains to the area design (Step 3) in the development process. The current study will provide knowledge on information and support needs people ask for when invited to CRC screening, which is important when designing the DA.

## 2 METHODS AND DESIGN

### 2.1 Design

This is a descriptive study encompassing data from documented telephone calls with two time periods during 2014–2018.

### 2.2 Setting

#### 2.2.1 The SCREESCO study

**Figure 1:**
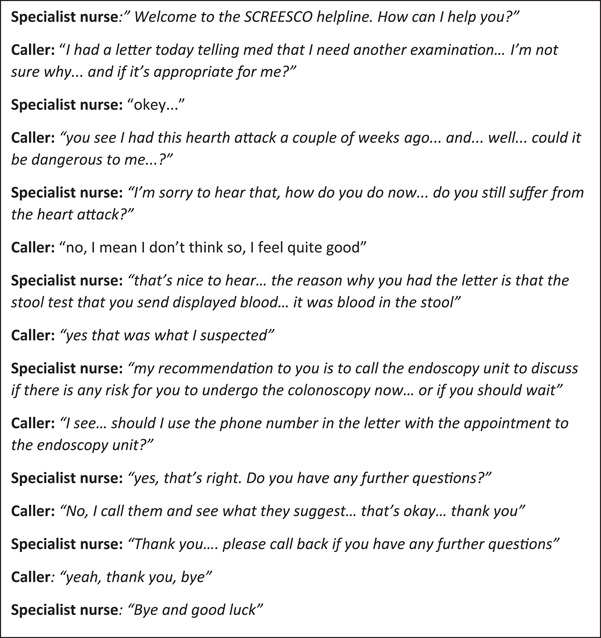
A fictive example of what a phone call to the SCREESCO helpline could look like. SCREESCO, Screening of Swedish Colons

Potential participants in the SCREESCO study^4^ were sent an invitation letter, with information about the study and the randomization result (i.e., colonoscopy or FIT). The letter included a telephone number and information about opening hours to a helpline where individuals could call when and if having questions. The helpline was open for 2 h all weekdays and staffed with experienced nurses (*n* = 3, whereof two are the authors K. F. and A. J.) and settled at the SCREESCO secretariat. A fictive example of a phone call is provided in Figure 1. The calls were documented during, or immediately after, in plain notebooks. There were no predefined questions or documentation templates.

#### 2.2.2 Study sample

For this paper, 2136 calls (21%) from two time periods were analysed: one at the beginning of the study (September 24, 2014 to March 16, 2015) and one at the end (September 20, 2017 to June 4, 2018).

### 2.3 Data analysis

The notebook data was transferred to predetermined categories in an Excel® sheet. The labelling of the categories was inspired by a similar study by Kirkegaard et al.^24^ The columns were filled in with a tick when appropriate, except for two columns with free text (reason for unsubscribing and counselling topics). One call could touch on several topics, that is, generating data on more than one column, and there is the possibility that the same individual called more than one time. The first author (K. F.) manually transferred relevant data to the Excel sheet and then, the first and last author (K. F., A. J.) compiled the data, checked for errors as far as possible and performed the analyses together.

The final Excel® sheet included information about: gender, randomization (FIT, colonoscopy), unsubscribing from screening, reasons for unsubscribing, confirm participation, logistical concerns related to the screening procedure, New screening kit/FIT difficulties, counselling and other. For an example of the categorization, please see Table 1. Reasons for unsubscribing were categorized into: Comorbidity, having had a previous colonoscopy, personal reason (e.g., lack of time), related to the screening method (e.g., wanted to change randomization group), having had a previous colectomy, unsubscribing from the first FIT round, feeling healthy, deceased and related to the laxative preparation. Counselling topics were categorized into: questions about FIT test/colonoscopy, dubiety about participation, anxiety and CRC and screening questions.

**Table 1:** An example of the categorization structure of documented calls

Gender	Randomization	Unsubscribing from screening	Reasons for unsubscribing	Confirm participation	Logistical concerns related to the screening procedure	Logistical concerns—commentary	New screening kit/FIT kit difficulties	Counselling	Counselling topics	Other
Male	Colonosopy	Yes								
Female	FIT	Yes	Negative to screening							
Female	Colonoscopy							Yes	Worry about the colonoscopy	
Male	Colonoscopy				Yes	No invitation letter				
Male	FIT (positive)							Yes	Recent myocardial infarction, insecure about the colonoscopy	

Descriptive data is being presented with proportions (*χ* ^2^ test applied) and group differences, such as between genders and randomization groups. IBM® SPSS® Statistics, Version 27 was used for the analyses and a *p*‐value of .05 was applied. Missing data was noted and is being presented in Section Results, 3.

### 2.4 Ethical approval

This study was ethically approved by the Swedish Ethical Review Authority (Dnr 2020‐02555).

## 3 RESULTS

Of about 90,000 invited individuals to either colonoscopy or FIT,^4^ around 10,000 calls (11%) were documented during the period from 2014 to 2020. Among the 2136 calls analysed for this paper, 1249 (58,5%) were from women and 1258 (59%) individuals were randomized to colonoscopy. No significant differences in randomization between men and women were found (*p* = .599). One or more reasons for calling were documented in 98% of the calls. Most frequent contents of the calls related to Unsubscribing from the study (25%) were followed by Confirmation of participation (24%), Logistical concerns about the screening procedure (e.g., booking and rebooking appointments for colonoscopy and questions about when to send in the FIT test) (20%), Counselling (19%) and New screening kit/FIT difficulties (10%). Comorbidity was the most frequent reason for unsubscribing (15%), followed by having had a previous colonoscopy (12%), personal reasons (9%) and the screening test itself (9%). However, 47% did not give any reason for unsubscribing and due to research ethics, the nurse was not allowed to ask for more information on the reasoning behind the decision. Most of the counselling calls were connected to the FIT test or the colonoscopy (57%), for example, how to perform the FIT test or questions about the upcoming colonoscopy, including how to prepare for it and the diet restrictions one should follow. Furthermore, 25% of the counselling calls concerned dubiety about participation, for example, related to comorbidity and/or current medication, to the individual's work situation or the distance to the screening centre or related to a general need to discuss pros and cons with CRC screening. Finally, 11% of the Counselling calls related to anxiety, for example, after being invited to the screening programme, after receiving an abnormal FIT test result or worries related to the colonoscopy and/or the laxative preparation.

A few differences in calling patterns between men and women were found, significantly more women called to unsubscribe from the study, while more men called to confirm participation. Differences in calling patterns were also found between those randomized to colonoscopy and to FIT regarding all reasons for calling the helpline. Individuals randomized to colonoscopy called to unsubscribe from the study or to confirm participation significantly more often while those randomized to FIT called significantly more often because of logistical concerns and/or counselling (Table 2).

**Table 2:** Reasons for calling the SCREESCO helpline

	*N*	Women *n* (%)	Men *n* (%)	*p* value	Colonoscopy *n* (%)	FIT *n* (%)	*p* value
Participants calling	2136	1249 (58)	887 (42)		1258 (59)	878 (41)	.599^a^
Unsubscribing from screening^b^	531	349 (28)	182 (21)	<.001	399 (32)	132 (15)	<.001
Confirm participation^b^	517	252 (20)	265 (30)	<.001	485 (39)	32 (4)	<.001
Logistical concerns related to the screening procedure^b^	420	253 (20)	167 (19)	.445	210 (17)	210 (24)	<.001
New screening kit/FIT difficulties^b^	223	128 (10)	95 (11)	.785	na^c^	na^c^	na^c^
Counselling^b^	410	256 (21)	154 (17)	.079	135 (11)	275 (31)	<.001

Abbreviations: FIT, faecal immunochemical test; na, not applicable; SCREESCO, Screening of Swedish Colons.

^a^ Tested between gender and randomization group.

^b^ Tested between those who called for the particular reason versus those who did not. Percentages are calculated within women/men and colonoscopy/FIT, respectively.

^c^ Differences not tested because ‘New screening kit/KIT difficulties’ not applicable for those randomized to colonoscopy.

## 4 DISCUSSION

To our knowledge, this is the first study to explore the calling patterns of a helpline from individuals invited to CRC screening in Sweden. Most calls were about unsubscribing, with comorbidity as the most frequently given reason, followed by confirming participation and calls about the logistics of the screening procedure. Counselling calls were less frequent and mainly related to the screening method. Women called to unsubscribe from the study more often than men, while men called to confirm participation to a higher extent. Those randomized to colonoscopy mainly called to unsubscribe from the study or confirm participation while logistic concerns and/or counselling were the main reasons for calling in those randomized to FIT. The findings including the significant differences between genders and randomization groups are of importance to bear in mind during the design of the DA. Given the findings that a considerable number of calls related to unsubscribing from the study or confirming participation (not actually required from the invited individuals) as well as logistical concerns, could relate to unclear information and/or that information should be presented in alternative ways. In one of our previous studies, participants and nonparticipants in the SCREESCO study described aspects such as: the invitation letter must draw one's attention, not contain too much text and also the importance of having multiple information sources,^19^ making our previous results and the present findings important in the DA development. Another recent study by Schwartz et al.^25^ focusing on lay people's views regarding the design of DAs for CRC screening, presented interesting and congruent results, that is stressing that a DA should be simple and short, tailored for different users and not contain heavy quantitative information. A well‐known principle of communication strategies is to keep messages short and easy to comprehend, however, not so commonly recognized in guidelines and literature on DAs.^25^ In addition, and in line with the Danish study,^24^ most of the counselling calls were due to a lack of knowledge of how to perform the FIT test or how to prepare for the colonoscopy. Those calls may have been preventable with easier access to information. Based on this, we find it important to further stress the tailoring part in our DA developing process, that is, provide limited information initially, with the possibility of access to more, if desired by the individual, but with respect to the individual's needs, health and digital literacy.

The total number of calls to the SCREESCO helpline was in line with the study from Denmark.^24^ Although the percentage of calls, in relation to all invited, can be viewed as low, the actual number of individuals calling is substantial and after having analysed calls from the second period it was evident that no new information was presented why the decision was made to not include additional notebooks. The calling pattern showed that many calls seemed to be related to a lack of understanding regarding the screening organization and screening methods, all of which could be dealt with in a DA. Still, considering the upcoming national screening programme in Sweden, including all individuals of a certain age group, and for that purpose suitable communication strategies—this study acknowledges the importance of having access to health care professionals since quite many used the helpline after all. This could pose a challenge in a country like Sweden, since our previous studies,^18^, ^21^ found that individuals make their screening decision on their own, without any involvement from the health care sector. However, one study^21^ showed that for those who discussed their decision, family, friends and fellow workers were most often approached, followed by nurses and physicians. The access could be solved through a helpline and a chat function included in the DA, with the possibility to discuss dubiety and anxiety, preferably with a specialized nurse as in the SCREESCO study, when deciding upon screening participation, or not.

The effectiveness of organized screening is directly linked to the participation rate. With a low participation follows a decreased impact on mortality and the cost to save one life increases.^26^ The aim of screening programmes is to improve health on a population basis, still not all invited individuals will benefit from taking part. There are associated risks with participation, such as false‐positive and false‐negative test results,^27^ as well as a risk with the procedure itself.^10^ Taken this into account, it is desirable not only to achieve a high uptake in CRC screening but a high uptake among people who have made a well‐informed decision. Therefore, the present findings together with three suggested areas,^26^ relating ethical issues to CRC screening, will be important when developing the DA. The first area is education, which will be approached here by providing information in the DA on CRC and screening, including the downsides of screening. The second area: applying diversifying approaches, will be addressed in the design and evaluation of the DA by approaching individuals who normally participate in screening to a lower extent, such as foreign‐born, individuals with disabilities and those living outside of society. The DA will continuously be evaluated to meet future demands, such as new scientific evidence, which pertains to the third area: the evolution of the screening programme.^26^

No connection between the existing SCREESCO register and the caller was done, limiting our possibilities to include additional clinical variables, which would have enabled us to perform more sophisticated analyses, such as logistic regressions. Furthermore, the fact that 47% of the callers did not give any reason for unsubscribing poses a limitation, but due to research ethics, the nurse was not allowed to ask for more information. Still both these aspects could have been planned for, beforehand, making a room for improvement when designing similar studies in the future. In addition, no particular protocol or checklist was used to document the calls, as Kirkegaard et al.^24^ did in their study. However, their labelling of categories inspired our analysis and turned out to be a useful template. Still, not using a predesigned template may increase an unbiased conversation between the nurse and the individual calling the helpline.

## 5 CONCLUSION

According to the results, many of the calls to the helpline seemed to be related to individuals' lack of understanding of the organization of the screening programme and the screening procedure. Therefore, we find it important to further stress the tailoring part in our DA developing process, that is, provide limited information initially, with the possibility of access to more, if desired by the individual, still with respect to the individual's needs, health and digital literacy.

## Data Availability

Data are available on request due to privacy/ethical restrictions.
